# Regional income convergence in Colombia: population, space, and long-run dynamics

**DOI:** 10.1007/s00168-022-01163-5

**Published:** 2022-08-10

**Authors:** Jesús Peiró-Palomino, William Orlando Prieto-Bustos, Emili Tortosa-Ausina

**Affiliations:** 1 Universitat de València and INTECO, Valencia, Spain; 2 Universidad Católica de Colombia, Bogotá, Colombia; 3 Universitat Jaume I, IIDL and Ivie, Castelló de la Plana, Spain

**Keywords:** C16, O18, O47, R11

## Abstract

We examine the trajectory of regional income dynamics in Colombia. Using data on all 33 Colombian departments from 2000 to 2016, we employ extensions of (spatial) Markov chains, space-time mobility measures, along with a fully weighted version of the distribution analysis approach. By considering these extensions, our analysis enables us to answer questions such as whether the role of spatial context influences the distributional dynamics of Colombian departments, or the magnitude of the moderating effect of department’s population. The inclusion of additional measures such as the asymptotic half-life of convergence provides additional results, informing on how long it would take to reach the hypothetical long-run distribution of per capita income. Results, which are reported for both pre- and post-2008 trends, complement previous literature on regional economic convergence in a relevant South American context, showing stronger convergence patterns when controlling for the population living in each department. The patterns do not particularly intensify when controlling for spatial spillovers, since the role of spatial context was already playing a relevant role from the beginning of the period analyzed. Therefore, although the ergodic distributions show a conditional-convergence pattern, addressing the problems of spatial exclusion fully, persistent polarization among geographical departments and populations, along with the relevant core-periphery gaps, still requires the design and implementation of specific policies.

## Introduction

Concerns about countries’ wealth have triggered a vast literature on growth and convergence. Conclusions as to the validity of the convergence hypothesis vary depending on methodologies, units of study (countries/regions), or sample years. The relevance of the issue has prompted a vast body of literature dealing with the topic, nicely reviewed by Islam (2003) and, more recently, Johnson and Papageorgiou (2020). Although most of this wave of research focused initially on international income convergence, regional convergence has become a large area in itself.

If we also factor in the growing inequality in income distribution in rapid-growth countries at a global level (Johnson and Papageorgiou 2020), these global tendencies would indicate that examining convergence at sub-national levels seems to be as important as at the country level. As Jerzmanowski (2006) indicate, over time, growth experiences differ within a country (almost) as much as they differ among countries (for recent relevant contributions see, for instance,Hierro et al. 2019; Marchand et al. 2020; Wei et al. 2020). Indeed, in some relevant regional contexts such as the European Union, the objective of convergence has involved specific policies (the so-called “cohesion policies”, see Farole et al. 2011; Bourdin 2019) and a large amount of economic resources (Sala-i-Martin 1996a; Giannetti 2002; Geppert and Stephan 2008; Ramajo et al. 2008). With a much more limited budget, this is also the case of some developing countries such as Colombia, the country on which we focus, and whose high levels of income disparities are a major concern among its policymakers.

Colombia is a highly unequal country with historical economic and social gaps due to disparities in human and physical capital, low-quality institutional settings and civil conflicts that have caused wealth inequities among and within regions (García and Benitez 1998; Galvis and Meisel 2010; Galvis-Aponte et al. 2017). It is well-known that great inequalities have an impact on redistributive tax pressures, deterring investment incentives and, ultimately, leading to more unstable socio-political environments with detrimental effects for economic activities (see, for instance, Alesina and Perotti 1996; Alesina and Rodrik 1994). Although Colombia is one of Latin America’s most solid performers in terms of economic growth over the last decades, this has not been felt equally throughout the country. In terms of population, the country is comparable to some developed countries such as Spain, but its regional inequalities are five times higher than those of the United States and Canada, and 42 times larger than in Australia (OECD 2014). These persistent regional disparities present a challenge and thwart the future development of the country, particularly in terms of *balanced* development (World Bank 2018).

Different regional convergence patterns can be distinguished from 1960 to the mid-2000s. There was a first period of convergence from 1960 to 1980, mainly driven by transport infrastructure investments (Bonet and Meisel 1999). This was followed by a period of divergence from 1980 to 1990, when the central region led economic development (Galvis and Meisel 2001; Acevedo 2003a). Finally, disparities persisted from 1990 onwards, when mobility between rich and poor regions was negligible (Bonet and Meisel 2008). The absence of economic convergence becomes a structural bottleneck, hindering equal opportunities for social and economic development in the country, while at the same time showing up poor performance of public policies in providing favorable conditions to push the lagged economies towards a sustainable growth pattern.

The reasons underlying these persisting regional imbalances are varied, and include the limited physical government presence in isolated regions, imbalances in essential public infrastructures, the country’s uneven topography (representing natural barriers isolating some areas, which remain disconnected), or the long armed conflict that has eroded the human, physical and even social capital of the most affected areas—particularly rural ones (World Bank 2018). However, given the limited performance of the more traditional neoclassical model to explain income dynamics among the Colombian regions, some of the pioneering contributions to the field such as Cárdenas (1993) or Cárdenas and Pontón (1995) suggested the need for alternative theories able to better explain the Colombian reality. As a result, endogenous growth models with increasing technological returns to scale based on human and physical capital spillovers were postulated as good candidates to explain the evolution of income convergence. In addition, geographical comparative advantages and demographic factors might have better capacity to explain the polarization patterns found. In this regard, more recent papers by Galvis et al. (2010) and Galvis-Aponte and Hahn-De-Castro (2016) have highlighted the role of spatial dependence and neighbor effects, which can be essential for the diffusion of the above-mentioned spillovers. Observed trends also reveal that fiscal policy decentralization has not been successful in closing per capita income gaps among central and peripheral regions in Colombia. In response, the new strategies for regional policy are based on a Regional Compensation Fund (RCF) to level up social and economic opportunities. The RCF is a long-term regional development policy proposal based upon human capital investments within a spatial and integrated approach designed to overcome an unequal wealth distribution (see Galvis et al. 2010).

Against this background, we examine the complexity of the convergence process in per capita income across Colombian departments over the period 2000–2016. The literature in this regard is already ample and has been recently reviewed by Galvis-Aponte et al. (2017). They document 20 years of studies evaluating different facets of regional convergence in Colombia, which vary in the methods used, periods considered or even variables assessed—not only economic magnitudes such as income per capita but also social variables. Because of these heterogeneities, results are not always entirely coincidental, but if the analysis is restricted to some specific methods they are generally more robust. In this study, and unlike several contributions that apply either $\sigma$- or $\beta$-convergence (which sometimes require strong assumptions), we follow the distribution approach initially developed by Quah (1993a, 1993b), which allows data to reveal the nature of the relationship of interest by using non-parametric techniques, and does not impose any assumption or restriction on the specification of the income distribution. In the analysis of Colombian regional convergence (we will discuss this question below), several studies consider the distribution approach have found that, in general, convergence has been weak—although results also vary depending on the period considered—and regional disparities persist.

These conclusions have been reached by Ardila Rueda (2004), Birchenall and Murcia (1997), Bonet and Meisel (2007), Branisa and Cardozo (2009b), Martínez (2006), Royuela and García (2015), Gómez (2006), all of whom consider different variants and instruments within the general context of the distribution approach. However, there are some gaps in this literature that we attempt to fill, and that constitute our contributions. First, although it is critical to evaluate intra-distribution mobility (i.e., changes in departments relative positions), very few contributions have measured it explicitly via transition probability matrices (Ardila Rueda 2004; Bonet and Meisel 2007). Although several studies consider internal mobility, represented by stochastic kernels, disregarding transition probability matrices constitutes an impediment to evaluate the ergodic (or stationary) distributions; we avoid this problem by also considering the continuous a state-space approach (Johnson 2005; Kremer et al. 2001). A further contribution we make to the convergence literature in Colombia is to explicitly measure intra-distribution mobility, by calculating mobility indices (in an analysis for an earlier period Birchenall 2001), as well as the asymptotic half-life of convergence—that is, we can answer questions as to when will the hypothetical stationary distribution be achieved. These are all relevant questions which to date remain either partly or wholly unanswered.

However, we consider an even more relevant contribution of the study to control explicitly for the role of demography and geography—two issues which, in the case of Colombia, are particularly pertinent. Considering demography, and taking into account population matters, convergence might be weak in purely *geographic* terms, but the patterns can differ when considering how many inhabitants populate each region—in our case, departments. As Sala-i-Martin (2006) notes, the unweighted approach is not useful if one is concerned about human welfare, since different regions have varying population sizes, and therefore a different share of the Colombian population living in poverty. This shift to population-weighted comparisons has obvious implications for the importance that we assign to the growth of the largest departments (Schultz 1998).

In turn, geographical features such as large mountain ranges and rain forest areas represent frictions that hinder connections and, therefore, make some areas more isolated. This can ultimately exacerbate regional disparities and heavily impact the convergence process. In the specific context of Colombia, only Galvis-Aponte and Hahn-De-Castro (2016) have partly dealt with these issues, although from a different point of view. As an additional contribution, the transition probability matrices enable the computation of ergodic (steady-state) distributions, which have not previously been considered for the case of Colombian regional convergence. We compute these distributions considering not only the unconditional analysis but also analyses for the two conditioning schemes (geography and demography), as well as their internal movements, following Johnson ’s (2005) proposals. The information provided by transition probability matrices is also complemented via the explicit measurement of intra-distribution mobility (Shorrocks 1978) and asymptotic half-life of convergence (Kremer et al. 2001), which tells us how far we are from reaching the ergodic distribution.

The results suggest that convergence in terms of GDP per capita is not taking place across Colombian departments in the analyzed period. In contrast, we observe a bimodal distribution, with a strong polarization between poor and rich departments that is more compatible with the concept of *club convergence*. This pattern changes when distributions are weighted by population. For that case, the resulting distribution is clearly unimodal and sharper than the unweighted one, showing a strong convergent process when we account for demography. Similarly, geography is also relevant, as convergence is much more evident when departments are compared with their neighbors than with the country mean.

The rest of the paper is organized as follows. Section 2 provides a short review of the related literature, whereas Sects. 3 and 4 are devoted to explain the methodology and describe the data. In Sect. 5 the results are presented and, finally, Sect. 6 concludes.

## Regional convergence in Colombia: the state of the art

There is a fairly large body of literature analyzing convergence in Colombia, either focusing on per capita income or other related economic or social variables, although some of the most relevant contributions were published before more recent important events, such as the international financial crisis or the end of the armed conflict. A review of the latest research on economic and social convergence in Colombia, either focusing on per capita income or other related economic or social variables, has mainly shown a polarized country, a situation that is persistent over time among departments (Galvis-Aponte et al. 2017).

Some of these studies, particularly the oldest ones, adopted $\sigma$ and $\beta$-convergence approaches. This is the case of Cárdenas and Pontón (1995) (see also Cárdenas 1993; Cárdenas et al. 1993), who evaluated per capita income convergence across departments for the 1950–1990 period, finding a robust convergent pattern. However, this result was not robust across studies, since other authors found convergence in the 1950–1960 period, but not for 1960–1990 (Meisel 1993). Similarly, Birchenall and Murcia (1997) and, to a lesser extent, Birchenall (2001), considered Quah’s distribution dynamics approach, finding weaker evidence supporting convergence. In another relevant study, Bonet and Meisel (2008), also using the distribution approach and with a new database, found that there was no clear pattern towards convergence between 1975 and 2000, and that Bogotá was playing a fundamental role in this process due to its size, both in population and economic terms.

Bonet and Meisel (1999) also found a significant negative relationship between initial income levels and growth rates and a reduction in the dispersion around the national income average from 1926 to 1960 due mainly to investment in roads and railways around the country. Nevertheless, the convergence trend changed from 1960 to 1995, when it showed a polarization in per capita income levels in which Bogotá was the dominant economic force in the country. The main factors behind the polarization process were the import substitution policy implemented to protect national industry and public consumption, which were more relevant in the capital city.

In turn, Rocha and Vivas (1998), Acevedo (2003b), Galvis and Meisel (2001), and Galvis-Aponte and Hahn-De-Castro (2016) showed how factors such as human and physical capital, market imperfections, political stability, international trade, telecommunications infrastructure, among others, matter when explaining regional growth. In this sense, there was a change in research focus, which shifted to the relevance of knowledge externalities together with increasing returns to scale to explain why some regions grew faster than others. In this new research trend, both these factors received particular attention, together with spatial dependence, spillovers and labor migration, effects that were included in econometric analyses. The results confirmed a higher concentration of economic activity, population and infrastructure in a few cities, located mostly in the central region. In contrast, peripheral regions are left behind, unable to close the regional income gap (Bonet 2007). Also, Ardila Rueda (2004) found that the decentralized fiscal policy has not been successful in promoting lower regional gaps. In this sense, regional public investment and regional public consumption only showed positive effects on the relative position of each region within the income distribution, but income distribution remained virtually unaltered between 1985 and 1996.

Other studies such as Galvis et al. (2010) found more evidence of convergence clubs (Phillips and Sul 2009) where income inequalities are lower compared to the distribution of all departments around the national average. They also found a polarization trend among convergence clubs, driven by spatial factors that are creating persistent poverty traps in peripheral regions. One of the most recent applications of the distribution dynamics approach (although they also considered $\sigma$ and $\beta$-convergence) to the case of the Colombia is the study by Royuela and García (2015), which analyzed not only the evolution of per capita income convergence, but extended the analysis to well-being indicators such as life expectancy, infant mortality, educational enrolment and crime issues. Their study, focusing on the period 1975–2005, found different patterns depending on the indicator considered. Although convergence was found for some social indicators (education, health, crime), per capita income exhibited a divergent pattern, a similar finding to Branisa and Cardozo (2009a) and Franco and Raymond (2009).^1^

## Methodology

Our methods build on the distribution approach initially proposed by Danny Quah in a series of contributions. With respect to other methods and concepts, particularly $\sigma$ and $\beta$-convergence, it has the advantage of analyzing how the entire distribution of per capita income evolves. Although, as mentioned in the preceding section, some contributions have already considered its application to the Colombian case, we introduce certain variations in the methodology that are novel in this context, and which provide more thorough conclusions. The advantages of analyzing the entire cross-sectional distribution of per capita income are multiple and include, for instance, a better ability to detect multi-modality, polarization, or the existence of convergence clubs (Phillips and Sul 2007).

Apart from choosing a methodology, any convergence study must take some additional decisions (Islam 2003). Some of them concern which *concept* of convergence to use; in our case we use convergence within an economy (Colombian departments), and GDP per capita-convergence. In addition, although in the first stages of the analysis we focus on *unconditional* (absolute) convergence, we will also examine several *conditioning* scenarios, by evaluating the role of geographic proximity, as well as the population size.

### On the shape of the distributions and their evolution: densities estimated via kernel smoothing and local polynomials

In the first stage of the model, we report the non-parametric estimation of per capita income density functions via kernel smoothing for different years. A concentration of the probability mass would indicate convergence, while flatter densities would indicate divergence. In addition, a multiplicity of scenarios could also emerge, such as the existence of convergence/divergence clubs (Ben-David 1994; Phillips and Sul 2009) shown by multi-modal shapes.

In our setting, where $x_{i,t}$ refers to department *i*’s normalized per capita GDP in period *t*, the corresponding kernel estimator will be:

1 $$\begin{aligned} {\hat{f}}(x)=\frac{1}{Nh}\sum \limits _{i=1}^N K \Big ( \frac{\Vert x-X_i\Vert _x}{h} \Big ) \end{aligned}$$

where *X* is departmental per capita income, *N* is the number of departments, *x* is the point of evaluation, $\Vert \cdot \Vert _x$ is a distance metric on the space of *X*, *h* is the bandwidth, and *K*(*x*) is a kernel function.^2^ The choice of the bandwidth has a much greater impact than the choice of kernel, however. We follow the local likelihood variant of density estimation, which allows us to overcome some notorious problems in kernel estimation (see Loader 1996; Hjort and Jones 1996).^3^ As Loader (1996) showed in his comparison of the relative efficiencies of kernel and local log-polynomial methods, the latter might perform better in settings such as ours, where several types of densities (unweighted, weighted, spatially conditioned, ergodic) are considered. Therefore, we consider changes in the local likelihood criterion as follows:

2 $$\begin{aligned} \sum \limits _{i=1}^{N} \omega _i (x) \text {ln}(f(X_i)) - N \int W \Big ( \frac{u-x}{h} \Big ) f(u) du \end{aligned}$$

where the log-link is used, i.e., $\text {ln}(f(x))$ is modelled by local polynomials, where *W* indicates that we specify a locally weighted least squares criterion for each fitting point (*x*), $\omega _i(x)$ refers to the localization weights, the log-link is used (i.e., $\text {ln}(f(x))$ is modeled via local polynomials), and the term on the right is the added penalty term.^4^ Despite the advantages of local bandwidth selection methods, and as we shall see, in order to assess the sensitivity of the results to the smoothing parameter, some of the kernel distributions were estimated using one of the best global alternatives: Sheather and Jones ’s plug-in bandwidth.^5^

### How densities evolve: intra-distribution mobility

Although two identical densities would in principle imply, neither convergence nor divergence, this could be concealing changes in departments’ relative positions—or *churning*. Therefore, apart from the evolution of the external shape of the distribution, it is also interesting to analyze its internal mobility. To do so, and considering our $x_{i,t}$ variable referring to department *i*’s normalized per capita GDP in period *t*, $F_{t}(x)$ is the cumulative distribution of $x_{i,t}$ across departments. A probability measure $\lambda _{t}((-\infty ,x])=F_{t}(x), \ \forall x \in {\mathbb {R}}$, $\lambda _{t}$ being the probability density function for each indicator across departments in period *t*.

We will look for the operator, $P^*$, that discloses information on how the distribution of per capita GDP at time $t-1$ transforms into a different distribution at time *t*. To do this, we focus on a stochastic difference equation $\lambda _t=P^*(\lambda _{t-1}, u_t), \ \text {integer} \ t$, which takes into account that $\{u_t: \text {integer} \ t \}$ is the sequence of disturbances of the entire distribution. In this context, $P^*$ is the operator mapping disturbances into probability measures, and which encodes the information on intra-distribution mobility. If we assume that operator $P^*$ is time invariant, and that the stochastic difference equation is of first order (Redding 2002), by setting null values to disturbances and iterating for $\lambda _t=P^*(\lambda _{t-1}, u_t)$ the future evolution of the distribution can be obtained, i.e., $\lambda _{t+\tau }=(P^*)^\tau \lambda _t$.

If the set of possible values of *x* is discretized into a finite number of classes (grids), to which we can also refer as states or intervals, $e_k$, $k \in \{1,\ldots ,K \}$, then $P^*$ will become a transition probability matrix as in:

3 $$\begin{aligned} \lambda _{t+1}=P^* \cdot \lambda _t \end{aligned}$$

We selected five states, which correspond to the quintiles of the relative income distribution. This is a reasonable choice as it takes into account representative parts of the distribution. The lowest and highest quintiles consider the poorest and richest regions, respectively. The second, third and fourth quintiles capture lower-medium, medium and upper-medium income departments, respectively. Accordingly, $\lambda _t$ turns into a $K \times 1$ vector of probabilities that the per capita GDP of a given department is located on a given grid at time *t*. It is then possible to evaluate the probability of a given department moving to a higher (or lower) position on the grid. We start by discretizing the set of observations into the states $e_{k}$.^6^ Each $p_{kl}$ entry in the matrix indicates the probability that a department initially in state *k* will transit to state *l* during the period (*T*) under analysis.

The limits between states are chosen so that all department-year observations are uniformly distributed among the cells. Other criteria for choosing the limits between states exist, including arbitrary (albeit ‘reasonable’) choices (Kremer et al. 2001; Quah 1993a). Accordingly, each cell in the transition probability matrices is computed by counting the number of transitions out of and into each cell. Therefore, each cell’s $p_{kl}$ value is:

4 $$\begin{aligned} p_{kl}=\frac{1}{T-1}\sum \limits _{t=1}^{T-1} \frac{n_{kl}^t}{n_k^t} \end{aligned}$$

where $n_{kl}^t$ is the number of departments moving during one period from state *k* to class *l*, $n_{k}^t$ is the total number of departments starting the period in state *k*, and *T* is the length of the sample period.

### Ergodic distributions, transition path analysis and mobility indices

The transition probability matrices allow us to characterize the ergodic or stationary distribution—under current trends. The resulting scenarios can be diverse, from distributions with the probability mass concentrated mainly in the central classes (indicative of convergence to the mean) to more polarized distributions with the probability mass distributed in the extreme classes (tails) of the distribution, indicating increasing separation between the poorest and richest, shown by twin peaks (Quah 1996b).

We compute the ergodic distributions following the algorithms proposed by Kremer et al. (2001). We also overcome the intrinsic disadvantages to transition probability matrices and ergodic distributions via transition probability matrices (i.e., the need to *discretize* per capita income into five states) by considering their continuous counterparts, following relevant proposals by Johnson (2000, 2005). This “continuous state approach”, theoretically developed by Johnson (2000, 2005), but also achievable empirically considering a sufficiently high number of states (Kremer et al. 2001), provides a continuous counterpart to the discrete ergodic distributions (based on transition matrices), with the advantage of not having to summarize information in a few states only.^7^

The ergodic distribution might not be reached quickly. Actually, it is unclear whether it would be ever reached, given it could only happen under current trends, which can vary. However, we can use the concept of asymptotic half-life of the chain ($H-L$), which refers to the time it takes to cover half of the distance to the ergodic distribution. We define the asymptotic half-life as:

5 $$\begin{aligned} H-L=-\frac{\text {ln} \ 2}{\text {ln} \ |\lambda _2|} \end{aligned}$$

where $|\lambda _2|$ is the second largest eigenvalue (after 1) of the transition probability matrix, ranging between infinity (when the stationary distribution does not exist and the second eigenvalue is equal to 1) and 0 (when $\lambda _2=0$ and the system has already reached its stationary equilibrium).^8^

In order to *quantify* the mobility underlying each transition matrix, we also consider mobility indices such as those considered in the economic inequality literature. Specifically, we follow Shorrocks (1978), Geweke et al. (1986) and Quah (1996a), some of whose proposals evaluate the trace of the transition probability matrix, providing information on the relative magnitude of on-diagonal and off-diagonal terms. Following Quah (1996a), its expression is:

6 $$\begin{aligned} \mu _1(P^*)=\frac{K-\text {tr}(P^*)}{K-1}=\frac{\sum _j (1-p_{jj})}{K-1} \end{aligned}$$

where $p_{jj}$ is the *j*-diagonal entry of matrix $P^*$, representing the probability of remaining in state *j*, and *K* is the number of classes. Large values of $\mu _1$ indicate more mobility (less persistence) in $P^*$. This concept is identical to the inverse of the harmonic mean of expected durations of remaining in a certain state.^9^

### Conditioning schemes: demography and geography

The methods presented in the previous sections provide a full analysis of departmental per capita income dynamics. However, departments differ widely in terms of population. In this section we propose a weighting scheme for the methods presented in the preceding paragraphs. We do this for both density functions as well as transition probability matrices, and the proposed weighting schemes can take several factors into account—in our case we will consider both population and space. The rationale for this is based on the relatively greater impact on per capita income convergence (or divergence) of a highly populated department than that of a sparsely populated one. One limitation of our conditioned approach is that it is not able to analyze inequality within departments. In other words, the fact that more populated departments are converging does not necessarily mean that the situation is improving for everyone. It can be argued, however, that we expect that more people are affected in largely populated departments. In addition, improvements in economic terms are usually accompanied by other aspects of social progress, which can ultimately reach more people. Hence, despite this limitation, taking into account the population size when analyzing convergence patterns can offer a better understanding of how many people are *potentially* affected. However, this issue has only rarely been taken into account in convergence studies applying the distribution dynamics approach, some exceptions being Tortosa-Ausina et al. (2005), Kremer et al. (2001) and Jones (1997).

Regarding the expressions corresponding to the non-parametric estimation of density functions, the modified kernel estimator becomes:

7 $$\begin{aligned} {\hat{f}}_{\omega }(x)=\frac{1}{h}\sum \limits _{i=1}^N \omega _i K \Big ( \frac{\Vert x-X_i\Vert _x}{h} \Big ) \end{aligned}$$

where $\omega _i$ corresponds to the share of Colombian population corresponding to department *i*. In our local likelihood approach for density estimation, the weights can be entered directly into Eq. (2).

Regarding the transition probability matrices, Equation (4) now takes into account the (potential) number of people that moves from one class to another. In this *weighted* transition probability matrix the expression corresponding to each cell will be:

8 $$\begin{aligned} p_{kl}^{\omega }=\frac{1}{T-1} \sum \limits _{t=1}^{T-1} \sum \limits _{i=1}^{n_{kl}} \frac{W_{ikl}^t}{W_{ik}^t} \end{aligned}$$

where $W_{ikl}^t$ is the population corresponding to department *i*, that moves from state *k* to state *l* in period *t*, and $W_{ik}^t$ is the population corresponding to department *i* starting the period in state *k*.

In turn, the effect of geography on convergence processes cannot be overlooked. Increasing returns to scale, knowledge spillovers, access to markets, labor mobility and vertical linkages between industries largely explain regional income and its geographical patterns. These issues have been widely explored, with particular intensity for the European regional context (see, for instance Breidenbach et al. 2019), although there are also some initiatives for the Colombian regional context such as Gómez Rodríguez and Santana Viloria (2016). The importance of explicitly taking spatial processes into account when assessing regional convergence has been repeatedly highlighted in the last years (Fischer and Stumpner 2008; Le Gallo and Fingleton 2019; Kelejian and Piras 2020). However, according to Gerolimetto and Magrini (2017), whereas convergence studies based on regression analysis devote a great deal of attention to the spatial phenomenon, within the nonparametric literature this issue has received much less attention.

In an attempt to address this issue, we conducted an analysis which compares the *state-relative* GDP per capita used in the previous sections and *neighbor-relative* per capita GDP, where we normalize each department’s per capita GDP by the average per capita GDP of the neighbor departments, excluding the department itself. The spatial econometrics literature provides many alternatives to define each department’s neighborhood, including distance, *k*-nearest criterion, a variety of economic and non-economic attributes or simply contiguity between two given regions. In this paper we follow this latter strategy, as contiguity matrices have been proved to capture spatial spillovers appropriately, while still being intuitive and simple in structure (LeSage 2014). Accordingly, those departments sharing borders are considered neighbors.^10^ Formally, the expression corresponding to the neighbor-relative per capita GDP series is:

9 $$\begin{aligned} x_i^{NR}=\frac{\text {ln} y_i }{\text {ln} \frac{1}{NE-1} (\sum _{j \in NE \setminus i} y_j)} \end{aligned}$$

where *NE* is the number of neighbors each *i* department has, and *nr* is the super-index indicating that we are referring to the neighbor-relative per capita GDP series. The closer the values of the neighbor-relative series are to unity, the lower the disparities among neighbor departments and the larger the magnitude of the spillover effects.^11^ As a robustness check, our baseline kernel distributions were computed using other additional neighboring criteria. Specifically, we considered the three, four and five closest regions (*k*-nearest criterion) as neighbors, and a distance-based alternative, which considers departments in a 300km radius as neighbors. This is the minimum distance to ensure that each region has at least one neighbor.

## Data and descriptive statistics

Two variables are used in the analysis: GDP per capita and population.^12^ Information on both variables was provided by the National Administrative Department of Statistics (DANE, *Departamento Administrativo Nacional de Estadística*). We consider the period 2000–2016.^13^ In contrast to other analyses considering previous periods, our selection allows us to consider all 33 Colombian departments—which, to our knowledge, has never been done before.^14^ The period considered is also novel, since with the exception of some analyses included in Galvis-Aponte et al. (2017), there is no evidence for the last 15 years and our analysis incorporates the computation of the ergodic distribution and conditioning schemes.

**Table 1:** Descriptive statistics, GDP$^{a}$ per capita, levels (*Y*/*N*) and annual growth rates (${\dot{y}}$)

Region/department	GDP per capita ($y=Y/N$)			GDP per capita annual growth rate, % (${\dot{y}}$)		
	2000	2008	2016	2000–2008	2009–2016	2000–2016
Andean region (*región Andina*)						
Antioquia	9.527	11.670	14.776	2.280	2.656	2.615
Bogotá	15.365	18.491	22.209	2.078	2.057	2.191
Boyacá	8.120	11.490	16.222	3.932	3.907	4.155
Caldas	6.426	8.835	10.604	3.601	2.048	2.990
Cundinamarca	9.257	10.975	13.500	1.909	2.328	2.244
Huila	7.328	8.838	10.534	2.104	1.970	2.158
Norte De Santander	5.370	6.992	8.648	2.975	2.390	2.842
Quindio	7.073	7.243	9.695	0.263	3.293	1.872
Risalarda	6.705	8.641	11.051	2.858	2.771	2.983
Santander	10.962	18.962	25.114	6.278	3.171	4.997
Tolima	6.579	8.902	10.595	3.417	1.953	2.843
Mean	8.428	11.003	13.904	2.882	2.595	2.899
Median	7.328	8.902	11.051	2.858	2.390	2.842
Standard deviation	2.817	4.119	5.363	1.512	0.639	0.928
Coefficient of variation	0.334	0.374	0.386	0.525	0.246	0.320
Caribbean and Insular regions (*regiones Insular y Caribe*)						
Atlántico	7.753	9.204	11.768	1.923	2.768	2.485
Bolívar	6.903	10.563	13.476	4.840	2.743	4.014
Cesar	5.955	10.839	12.346	6.880	1.457	4.382
Córdoba	5.350	6.301	7.103	1.833	1.341	1.681
La Guajira	6.300	8.708	6.441	3.663	− 3.296	0.130
Magdalena	4.332	5.740	7.022	3.175	2.266	2.882
San Andrés	8.598	10.581	13.640	2.332	2.862	2.752
Sucre	3.970	5.008	6.551	2.616	3.029	2.991
Mean	6.145	8.368	9.793	3.408	1.646	2.665
Median	6.128	8.956	9.435	2.896	2.505	2.817
Standard deviation	1.599	2.364	3.283	1.719	2.097	1.328
Coefficient of variation	0.260	0.282	0.335	0.505	1.274	0.499
Pacific region (*región del Pacífico*)						
Chocó	2.873	3.895	5.891	3.439	4.705	4.314
Valle del Cauca	10.119	12.031	14.498	1.942	2.093	2.138
Cauca	4.054	5.677	8.878	3.812	5.093	4.718
Nariño	3.821	4.845	6.365	2.671	3.079	3.047
Mean	5.217	6.612	8.908	2.966	3.743	3.554
Median	3.938	5.261	7.621	3.055	3.892	3.680
Standard deviation	3.308	3.686	3.950	0.831	1.404	1.183
Coefficient of variation	0.634	0.557	0.443	0.280	0.375	0.333
Orinoco region (*región de la Orinoquía*)						
Meta	10.186	18.702	21.176	6.984	1.390	4.399
Vichada	4.838	5.557	5.073	1.550	− 1.006	0.279
Casanare	45.042	30.441	24.332	− 4.260	− 2.458	− 3.558
Arauca	15.377	24.904	12.878	5.504	− 7.066	− 1.038
Mean	18.861	19.901	15.865	2.444	− 2.285	0.021
Median	12.782	21.803	17.027	3.527	Â1.732	− 0.379
Standard deviation	17.977	10.698	8.665	5.024	3.560	3.325
Coefficient of Variation	0.953	0.538	0.546	2.055	− 1.558	160.442
Amazon region (*región Amazónica*)						
Amazonas	4.603	5.056	6.387	1.049	2.631	1.946
Caquetá	4.145	5.051	6.872	2.222	3.479	3.019
Guainía	4.597	4.518	5.376	− 0.194	1.951	0.924
Guaviare	4.744	4.780	5.217	0.085	0.977	0.561
Putumayo	4.574	6.308	6.762	3.637	0.775	2.327
Vaupés	3.442	3.197	4.205	− 0.817	3.092	1.185
Mean	4.351	4.818	5.803	0.997	2.151	1.660
Median	4.585	4.916	5.881	0.567	2.291	1.565
Standard deviation	0.489	1.005	1.047	1.674	1.113	0.932
Coefficient of variation	0.112	0.209	0.180	1.680	0.517	0.562
Full sample (6 natural regions/33 departments)						
Mean	8.009	9.786	11.067	2.624	1.832	2.347
Median	6.426	8.708	10.534	2.616	2.328	2.615
Standard deviation	7.317	6.195	5.643	2.219	2.336	1.742
Coefficient of variation	0.914	0.633	0.510	0.846	1.276	0.742

$^{a}$ In millions of constant 2010 Colombian pesos

Source: National Administrative Department of Statistics (DANE, *Departamento Administrativo Nacional de Estadística*) and the authors

**Table 2:** Descriptive statistics for departments and regions, population$^{a}$, levels (*N*) and annual growth rates (${\dot{N}}$)

Region/department	Population (*N*)			Population annual growth rate (${\dot{N}}$)		
	2000	2008	2016	2000–2008	2009–2016	2000–2016
Andean region (*región Andina*)						
Antioquia	5.290	5.911	6.535	1.242	1.120	1.251
Bogotá	6.303	7.155	7.980	1.419	1.220	1.398
Boyacá	1.235	1.263	1.278	0.256	0.130	0.204
Caldas	0.959	0.974	0.990	0.173	0.175	0.184
Cundinamarca	2.077	2.398	2.721	1.608	1.418	1.603
Huila	0.938	1.054	1.169	1.306	1.152	1.301
Norte De Santander	1.190	1.276	1.368	0.782	0.776	0.825
Quindio	0.520	0.544	0.569	0.498	0.499	0.528
Risalarda	0.870	0.914	0.957	0.553	0.513	0.565
Santander	1.905	1.990	2.071	0.483	0.447	0.492
Tolima	1.337	1.379	1.412	0.346	0.266	0.324
Mean	2.057	2.260	2.459	0.788	0.701	0.789
Median	1.235	1.276	1.368	0.553	0.513	0.565
Standard deviation	1.826	2.089	2.348	0.513	0.458	0.514
Coefficient of variation	0.888	0.924	0.955	0.652	0.653	0.652
Caribbean and Insular regions (*regiones Insular y Caribe*)						
Atlántico	2.017	2.255	2.490	1.246	1.105	1.245
Bolívar	1.793	1.938	2.122	0.867	1.016	0.997
Cesar	0.845	0.941	1.041	1.212	1.128	1.239
Córdoba	1.362	1.535	1.736	1.343	1.375	1.440
La Guajira	0.549	0.763	0.985	3.735	2.876	3.502
Magdalena	1.119	1.180	1.272	0.593	0.840	0.759
San Andrés	0.068	0.072	0.077	0.717	0.738	0.770
Sucre	0.735	0.795	0.860	0.880	0.877	0.930
Mean	1.061	1.185	1.323	1.324	1.244	1.360
Median	0.982	1.061	1.157	1.046	1.060	1.118
Standard deviation	0.607	0.658	0.719	1.010	0.689	0.898
Coefficient of variation	0.572	0.555	0.543	0.763	0.554	0.660
Pacific region (*región del Pacífico*)						
Chocó	0.437	0.467	0.505	0.733	0.872	0.850
Valle del Cauca	3.949	4.294	4.661	0.934	0.916	0.979
Cauca	1.216	1.298	1.392	0.726	0.781	0.798
Nariño	1.446	1.600	1.766	1.125	1.105	1.181
Mean	1.762	1.914	2.081	0.879	0.918	0.952
Median	1.331	1.449	1.579	0.834	0.894	0.915
Standard deviation	1.317	1.435	1.558	0.190	0.136	0.170
Coefficient of variation	0.747	0.749	0.749	0.216	0.148	0.179
Orinoco region (*región de la Orinoquía*)						
Meta	0.697	0.836	0.980	2.027	1.785	2.019
Vichada	0.049	0.060	0.074	2.392	2.219	2.442
Casanare	0.264	0.313	0.363	1.927	1.636	1.887
Arauca	0.216	0.241	0.265	1.246	1.048	1.215
Mean	0.307	0.363	0.420	1.898	1.672	1.891
Median	0.240	0.277	0.314	1.977	1.710	1.953
Standard deviation	0.239	0.288	0.339	0.478	0.484	0.509
Coefficient of variation	0.781	0.794	0.807	0.252	0.289	0.269
Amazon region (*región Amazónica*)						
Amazonas	0.062	0.070	0.077	1.396	1.027	1.283
Caquetá	0.399	0.436	0.484	1.010	1.151	1.145
Guainía	0.032	0.037	0.042	1.780	1.426	1.698
Guaviare	0.089	0.100	0.113	1.322	1.306	1.392
Putumayo	0.294	0.319	0.350	0.943	1.007	1.033
Vaupés	0.036	0.040	0.044	1.312	0.904	1.173
Mean	0.152	0.167	0.185	1.294	1.137	1.287
Median	0.076	0.085	0.095	1.317	1.089	1.228
Standard deviation	0.142	0.154	0.170	0.300	0.198	0.236
Coefficient of variation	0.935	0.921	0.920	0.232	0.174	0.183
Full sample (6 natural regions/33 departments)						
Mean	1.221	1.347	1.477	1.155	1.056	1.171
Median	0.870	0.941	0.990	1.125	1.027	1.173
Standard deviation	1.416	1.586	1.757	0.693	0.550	0.654
Coefficient of variation	1.160	1.178	1.189	0.600	0.521	0.558

$$^{a}$$ In millions

Source: National Administrative Department of Statistics (DANE, *Departamento Administrativo Nacional de Estadística*) and the authors

Summary statistics are reported in Tables 1 and 2. The different columns provide information for income per capita (Table 1) and population (Table 2), both in levels as well as in growth rates. The information is split for selected years and periods. The period of analysis is 2000–2016, for a variety of reasons. Although, ideally, a longer period of analysis would have been more informative, particularly for the sake of comparison with previous literature, this would have impeded using all 33 departments in which Colombia’s territory is organized today. When deliberating this trade-off (i.e., having to choose either more years or more departments) we chose to drop some years in order to focus on the 33 departments, for which analyses of convergence are scarce.

In contrast to other recent convergence studies for Colombia, such as Acevedo (2003a), Ardila Rueda (2004), and Galvis and Meisel (2012), the following description includes all 33 departments, which is a relevant contribution to regional income distribution research in this context. Although available data would give us greater insight into distributional patterns at the municipal level, our key variable, per capita income, is not available at this level of disaggregation.

Table 1 reports data on per capita income and population in 2010 Colombian pesos per person for years 2000, 2008 and 2016, along with growth rates for the three subperiods considered (2000–2016, 2000–2008 and 2009–2016). There are remarkable discrepancies among both departments and natural regions.^15^ For instance, for the whole country, the year 2000 average for GDP per capita was about 8 million Colombian pesos per person (in 2010 pesos). Between 2000 and 2016, the per capita income grew in real terms at an annual rate of 2.3% to reach a level of 11 million per person (in 2010 pesos). Although all regions did show favorable growth rates in per-capita income, the Andean (*región Andina*) and Orinoco (*región de la Orinoquía*) regions exhibited the highest levels of per capita GDP. In contrast, the highest per capita income growth rates corresponded to the Pacific Region, which has been considered the country’s poorest natural region. Moreover, the Caribbean (*región Caribe*) and Andean regions also showed higher growth rates than the Orinoco region, which has the most important oil reserves in the country.

Having better growth rates in regions that are not endowed with natural resources, and having higher growth rates in poor regions are usually considered as evidence of per capita income convergence in terms of the traditional neoclassical approach (Rocha and Vivas 1998). However, some work using non-parametric methods (Bonet and Meisel 1999) has found a polarization process in per capita income levels, according to which the capital city, Bogotá, was the dominant economic force. Indeed, there is evidence of a reduction in the coefficient of variation in per capita income levels for each sub-period, but it happened simultaneously with an increase in per capita income growth dispersion for each sub-period.

Although the Andean and Orinoco regions had the best performance in terms of real per capita GDP levels, they also exhibited the most volatile evolution of the per capita GDP trend in each period, as shown by the standard deviation. In contrast, the coefficient of variation for the Amazon region almost doubled between 2000 and 2016—as some departments grew much faster than others—, a similar trend to that observed in the Caribbean region. In the Pacific region, however, another relatively poor area, per capita income grew much faster (it almost doubled between 2000 and 2016) than its dispersion and, as a consequence the coefficient of variations declined sharply. These descriptive findings show that there was a convergence process with winners and losers that might have offset the initial positive trend in terms of per capita GDP levels and growth rates for all regions.

In this sense, it is clear that the Andean region, particularly Bogotá and the department of Antioquia, are winners compared to the departments in the Pacific and Amazon regions. In all, performance of the departments of Antioquia, Valle del Cauca, and the capital city, Bogotá, which enjoy better transportation and communication infrastructures (and also better access to finance), improved in relation to regional and national averages. This descriptive finding reveals that the most developed departments of Antioquia, Valle del Cauca, and the city of Bogotá, partly driven by higher investments in public infrastructures and urbanization processes, and also due to labor migration patterns that have attracted people to these areas of the country.

A closer look at the data reveals a population trend that has a bearing on our understanding of migration patterns driven by the economic outlook in each province. The Andean region, particularly the departments of Antioquia and Bogotá, and the department of Valle del Cauca (in the Pacific region), have the highest population levels in the sample for each of the periods considered (see Table 2). However, the Orinoco and Caribbean regions have the highest population growth rates. In contrast, the Pacific and Amazon regions do not show an increase in population growth and remain the regions with the lowest population levels. This finding seems to be related to better employment opportunities and the presence of an urbanization process that had taken place in the economic triangle comprising the departments of Antioquia, Valle del Cauca, and the city of Bogotá. At the same time, the oil industry is also an important factor in explaining the positive population growth in the Orinoco region. There was also an economic diversification process that helped to increase the financial incentives to migrate from rural areas to urban areas, which is the case of the Caribbean and Andean regions. While Bogotá and the departments of Antioquia and Valle del Cauca dominate in terms of population *trends* (growth), the departments of Atlántico and Bolívar show the most significant population *levels* in the Caribbean region.

**Fig. 1:**
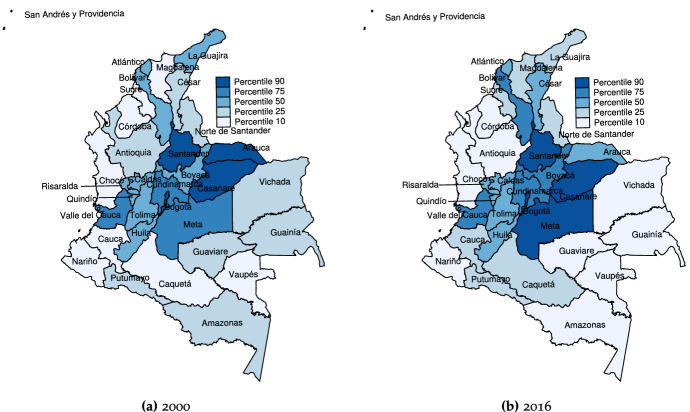
GDP per capita, Colombian departments, 2000 vs. 2016

The figures reported in the table give a clear idea of the remarkable discrepancies among departments and regions in terms both of wealth and population. Figure 1 provides a more visual picture in the form of a map of Colombian departments in which the lightest colors indicate lower per capita GDP, and the darkest colors, the highest. Here we see that more wealth is concentrated in the central areas (Orinoco and Andean regions), whereas the periphery (Pacific, Amazon and Caribbean regions) is not only poorer but actually grows poorer over time—the colors corresponding to the Amazon region, for instance, have become lighter in general. Therefore, wealth discrepancies are not only high but, as documented in previous literature, persistent. We disentangle these trends in the following sections.

**Table 3:** Convergence trends for Colombian departments (descriptive), normalized GDP per capita,$^{a}$ levels ($y/{\bar{y}}$)

Region/department	2000	2008	2016	Trend
Andean region (región Andina)				
Antioquia	1.190	1.193	1.335	Divergence (forging ahead)
Bogotá	1.918	1.890	2.007	Divergence (forging ahead)
Boyacá	1.014	1.174	1.466	Divergence (forging ahead)
Caldas	0.802	0.903	0.958	Convergence (catching up)
Cundinamarca	1.156	1.122	1.220	Divergence (forging ahead)
Huila	0.915	0.903	0.952	Convergence (catching up)
Norte De Santander	0.670	0.714	0.781	Convergence (catching up)
Quindio	0.883	0.740	0.876	Divergence (forging ahead)
Risalarda	0.837	0.883	0.999	Convergence (catching up)
Santander	1.369	1.938	2.269	Divergence (forging ahead)
Tolima	0.821	0.910	0.957	Convergence (catching up)
Caribbean and Insular regions (regiones Insular y Caribe)				
Atlántico	0.968	0.941	1.063	Divergence (forging ahead)
Bolívar	0.862	1.079	1.218	Divergence (forging ahead)
Cesar	0.744	1.108	1.116	Divergence (forging ahead)
Córdoba	0.668	0.644	0.642	Divergence (falling behind)
La Guajira	0.787	0.890	0.582	Divergence (falling behind)
Magdalena	0.541	0.587	0.634	Convergence (catching up)
San Andrés	1.074	1.081	1.232	Divergence (forging ahead)
Sucre	0.496	0.512	0.592	Convergence (catching up)
Pacific region (región del Pacífico)				
Chocó	0.359	0.398	0.532	Convergence (catching up)
Valle del Cauca	1.263	1.229	1.310	Divergence (forging ahead)
Cauca	0.506	0.580	0.802	Convergence (catching up)
Nariño	0.477	0.495	0.575	Convergence (catching up)
Orinoco region (región de la Orinoquía)				
Meta	1.272	1.911	1.913	Divergence (forging ahead)
Vichada	0.604	0.568	0.458	Divergence (falling behind)
Casanare	5.624	3.111	2.199	Convergence
Arauca	1.920	2.545	1.164	Convergence
Amazon region (región Amazónica)				
Amazonas	0.575	0.517	0.577	Convergence (catching up)
Caquetá	0.518	0.516	0.621	Convergence (catching up)
Guainía	0.574	0.462	0.486	Divergence (falling behind)
Guaviare	0.592	0.488	0.471	Divergence (falling behind)
Putumayo	0.571	0.645	0.611	Convergence (catching up)
Vaupés	0.430	0.327	0.380	Divergence (falling behind)

$^{a}$ To ease interpretation, this normalization corresponds to GDP per capita divided by the national average

Source: National Administrative Department of Statistics (DANE, *Departamento Administrativo Nacional de Estadística*) and the authors

**Table 4:** Convergence trends for Colombian regions (descriptive), normalized GDP per capita,$^{a}$ levels ($y/{\bar{y}}$)

Region/department	2000	2008	2016	Trend
Andean region (región Andina)	1.052	1.124	1.256	Divergence (forging ahead)
Caribbean and Insular regions (regiones Insular y Caribe)	0.767	0.855	0.885	Convergence (catching up)
Pacific region (región del Pacífico)	0.651	0.676	0.805	Convergence (catching up)
Orinoco region (región de la Orinoquía)	2.355	2.034	1.434	Convergence
Amazon region (región Amazónica)	0.543	0.492	0.524	Divergence (falling behind)

$^{a}$ To ease interpretation, this normalization corresponds to GDP per capita divided by the national average

Source: National Administrative Department of Statistics (DANE, *Departamento Administrativo Nacional de Estadística*) and the authors

Table 3 provides an initial view of the convergence trends for departments for the analyzed period, comparing years 2000, 2008 and 2016. In comparison to Table 1, it reports normalized (divided by the average for all departments) GDP per capita values. The last column reports the convergence trend for each department, contemplating the possibility not only of converging or diverging, but also other specific scenarios. When a divergence trend is identified, we can label it as “forging ahead” or “lagging behind”, depending on whether the department’s normalized per capita income is becoming increasingly higher or lower than the country’s average, respectively. The convergence scenario is simpler, since we contemplate the possibility of “catching up” (lagged departments that are closer to the national average by 2016), or simply “convergence” (richer regions whose initial advantage has been sliced by 2016). This descriptive analysis indicates that the cases of divergence (18) are more frequent than those of convergence (15), a result that corroborates some of the previous findings by the literature on Colombian regional convergence. The higher or lower cohesion is not overwhelmingly concentrated in specific regions but, as indicated in Table 4, which reports an analogous analysis for the Colombia’s natural regions, divergence is higher in the Andean region, which clearly forges ahead, and the Amazon region, whose per capita income is half the national average, and is almost stagnant comparing 2000 and 2016.

## Results

**Table 5:** Convergence across Colombian departments (per capita income, *GDP*/*N*), intra-distribution mobility and long-run trends

(Number of observations)	Upper limit, all years				
	0.970	0.988	1.005	1.023	Max.
(105)	0.81	0.19			
(92)	0.20	0.68	0.12		
(103)		0.10	0.73	0.17	
(99)			0.14	0.78	0.08
(96)				0.08	0.92
Ergodic distribution	0.10	0.10	0.12	0.28	0.41
Mobility index ($\mu _1$)	0.623				
Half-life of convergence	47.874				
(a) 2000–2016					
	Upper limit, all years:				
(Number of observations)	0.970	0.989	1.006	1.024	Max.
(45)	0.89	0.11			
(47)	0.18	0.72	0.09		
(47)		0.06	0.74	0.20	
(44)			0.14	0.78	0.07
(48)				0.06	0.94
Ergodic distribution	0.04	0.02	0.05	0.14	0.75
Mobility index ($\mu _1$)	0.624				
Half-life of convergence	72.024				
(b) 2000–2008					
	Upper limit, all years:				
(Number of observations)	0.970	0.988	1.004	1.020	Max.
(50)	0.80	0.20			
(43)	0.14	0.74	0.12		
(47)		0.09	0.74	0.17	
(46)		0.05	0.10	0.71	0.14
(45)				0.12	0.88
Ergodic distribution	0.19	0.26	0.18	0.20	0.16
Mobility index ($\mu _1$)	0.624				
Half-life of convergence	31.588				
(c) 2008–2016					

	Upper limit, all years				
(Number of observations)	0.970	0.989	1.006	1.024	Max.
(45)	0.89	0.11			
(47)	0.18	0.72	0.09		
(47)		0.06	0.74	0.20	
(44)			0.14	0.78	0.07
(48)				0.06	0.94
Ergodic distribution	0.04	0.02	0.05	0.14	0.75
Mobility index ($\mu _1$)	0.624				
Half-life of convergence	72.024				
(b) 2000–2008					
	Upper limit, all years:				
(Number of observations)	0.970	0.988	1.004	1.020	Max.
(50)	0.80	0.20			
(43)	0.14	0.74	0.12		
(47)		0.09	0.74	0.17	
(46)		0.05	0.10	0.71	0.14
(45)				0.12	0.88
Ergodic distribution	0.19	0.26	0.18	0.20	0.16
Mobility index ($\mu _1$)	0.624				
Half-life of convergence	31.588				
(c) 2008–2016

**Table 6:** Population-weighted convergence across Colombian departments, intra-distribution mobility and long-run trends

	Upper limit, all years:				
(Share of population)	0.970	0.989	1.006	1.024	Max.
(0.07)	0.81	0.19			
(0.12)	0.16	0.73	0.11		
(0.16)		0.09	0.77	0.13	
(0.28)			0.09	0.83	0.08
(0.37)				0.06	0.94
Ergodic distribution	0.02	0.05	0.08	0.31	0.53
Mobility index ($\mu _1$)	0.549				
Half-life of convergence	46.854				
a) 2000–2016					
	Upper limit, all years:				
(Share of population)	0.970	0.989	1.006	1.024	Max.
(0.07)	0.86	0.14			
(0.10)	0.12	0.78	0.10	0.00	
(0.16)		0.07	0.77	0.16	
(0.21)			0.10	0.77	0.13
(0.45)				0.05	0.95
Ergodic distribution	0.01	0.03	0.13	0.26	0.57
Mobility index ($\mu _1$)	0.574				
Half-life of convergence	158.355				
b) 2000–2008					
	Upper limit, all years:				
(Share of population)	0.970	0.988	1.004	1.020	Max.
(0.06)	0.74	0.26			
(0.14)	0.14	0.79	0.07		
(0.15)		0.11	0.74	0.15	
(0.30)		0.01	0.12	0.74	0.14
(0.35)				0.13	0.87
Ergodic distribution	0.03	0.12	0.12	0.27	0.46
Mobility index ($\mu _1$)	0.606				
Half-life of convergence	65.998				
c) 2008–2016

**Table 7:** Spatially conditioned convergence across Colombian departments, intra-distribution mobility and long-run trends

	Upper limit, all years:				
(Number of observations)	0.977	0.988	0.999	1.008	Max.
(101)	0.80	0.18	0.01	0.01	
(101)	0.18	0.69	0.13		
(95)		0.14	0.65	0.19	0.02
(98)			0.17	0.73	0.10
(100)			0.01	0.13	0.86
Ergodic distribution	0.16	0.18	0.20	0.26	0.20
Mobility index ($\mu _1$)	0.631				
Half-life of convergence	16.501				
a) 2000–2016					
	Upper limit, all years:				
(Number of observations)	0.979	0.989	0.999	1.010	Max.
(46)	0.88	0.10	0.00	0.02	
(46)	0.13	0.73	0.14	0.00	
(50)		0.12	0.61	0.22	0.05
(43)			0.15	0.73	0.11
(46)				0.15	0.85
Ergodic distribution	0.23	0.11	0.14	0.33	0.18
Mobility index ($\mu _1$)	0.634				
Half-life of convergence	24.629				
b) 2000–2008					
	Upper limit, all years:				
(Number of observations)	0.977	0.987	0.999	1.007	Max.
(73)	0.83	0.17			
(18)	0.29	0.38	0.28	0.06	
(23)		0.17	0.49	0.34	
(32)		0.06	0.18	0.69	0.06
(85)			0.02		0.98
Ergodic distribution	0.21	0.09	0.11	0.09	0.50
Mobility index ($\mu _1$)	0.680				
Half-life of convergence	24.068				
c) 2008–2016					

The results in the preceding section, albeit interesting, provide an initial and descriptive view of the regional per capita income trends in Colombia between 2000 and 2016. In this section we provide a fuller view, expanding the previous findings in the literature. Specifically, we provide results for all methods described in Sect. 3, including transition probability matrices, ergodic (stationary) distributions, mobility indices and asymptotic half-life convergence. We also report density functions, as well as results for the different conditioning schemes—population-weighted and physically contiguous conditioned. For the transition probability matrices, we present tables for the different periods and sub-periods considered (2000–2016, 2000–2008 and 2008–2016), for the unweighted analysis (Table 5), population-weighted (Table 6), and physically contiguous conditioned (Table 7). The last three rows in each panel display information on the initial, final and ergodic distributions of (normalized) departmental per capita income.

The variable of analysis is the normalized logarithm of per capita GDP. We normalize by dividing per capita GDP of department *i* in year *t* by that year’s national average, i.e., $x_{it}= \text {ln} y_{it}/\text {ln} {\bar{y}}_t$, where $y_{it}$ is the per capita GDP of department *i* in year *t*, and ${\bar{y}}_t$ is the cross-sectional average of $y_{it}$. By normalizing the data, we can assess more easily how far a given department is from the rest of the country—the closer a given (normalized) value is to unity, the closer it will be to the national average. This naturally implies that the more values closer to unity, the faster the convergence to the national average.^16^

### Unweighted distribution dynamics

Transitions for normalized departmental per capita GDP are reported in Table 5. The top panel reports results for the entire period (2000–2016), and the middle and bottom panels, for each sub-period (2000–2008 and 2008–2016, respectively). Since our period of analysis is not particularly long, we consider two-year overlapping transitions—i.e., from 2000 to 2002, from 2001 to 2003, and so on.^17^

For each of the matrices in Table 5, the cut-off points (upper limits) differ slightly because the period analyzed is different. Although several criteria are available, one of the most widely accepted is to consider all observations for the analyzed period (2000–2016, 2000–2008 or 2008–2016), and divide them into five similarly sized intervals. Accordingly, the numbers in brackets to the left of each matrix correspond to the number of observations (departments) starting the period in a given state (or class). In the case of the upper panel in Table 5, given we are considering two-year transitions, they sum to 495 (instead of 528), since the last two years (2015 and 2016) would be excluded (i.e., $495 = 33 \ \text {departments} \times 15 \ \text {transitions}$).

The first row of each panel displays the cut-off points that delimit the intervals (upper limits) and should be interpreted as follows: the upper limit for the first state of 0.970 implies that approximately one fifth of the total number of observations lie below 97% of the average (in logs). For the other tail of the distribution, the upper-state has observations above 1.023 (102.3%) of the average (in logs). Although this is a relatively narrow range of variation, note that the average is unity, since our data have been normalized by the mean, after taking logs.

Inside each $5 \times 5$ matrix in Table 5, entries (cells) should be interpreted as the probability of *remaining* in a particular state after two years—since we are considering 2-year transitions. For instance, in the case of the entire 2000–2016 period (top panel in Table 5), its value would indicate that 81% of the observations starting in the lowest relative per capita GDP state (105 observations, below 0.970) would remain in that state, whereas the remaining 19% would move to states of higher relative per capita income—in this case, to state 2. This high persistence is greater for richer departments, as shown by the probability in the lower right of the matrix, which shows that 92% of the observations in the richest state remain there after two years—with 8% moving to state 4. The rest of the values on the main diagonal show less persistence. Actually, the higher the probability off the main diagonal, the higher the mobility, whereas values on the main diagonal closer to one indicate more persistence.

Regarding the implicit mobility shown in Table 5, the values on the main diagonals of each matrix average to 0.784, 0.814 and 0.774 (for 2000–2016, 2000–2008 and 2008–2016, respectively), which suggests that most changes in the relative positions took place during the most recent period. These average values represent a good starting point to measure mobility. However, we can consider more precise measures which are less frequently used in distribution dynamics studies such as the mobility indices presented in Sect. 3.3.

Each of the three panels in Table 5 reports results for mobility indices (right below each of the row containing the ergodic distributions). They do not entirely corroborate what was found for the average values on the main diagonal, since $\mu _1$ shows quite similar values for the three periods. However, apart from the absolute value found for mobility, it is important to assess its implicit trends—i.e., whether it leads to convergence, divergence or other possible outcomes.

Specifically, each panel in Table 5 also reports information on the ergodic (steady-state) distributions for the selected periods. According to Table 5.a, under 2000–2016 trends, intra-distribution mobility drives probability mass to concentrate in the states of relatively high per capita income—with 69% of probability mass concentrated in states 4 and 5, and only 20% in the poorest states (1 and 2). This process of convergence to richer states, however, is the result of different dynamics, as shown in the central and bottom panels in the Table, since intra-distribution mobility in the first sub-period (2000–2008) leads probability mass to concentrate strongly (75%) in state 5. In contrast, under 2008–2016 trends (Table 5c), although convergence still existed, it was more concentrated in poorer states—with state 2 absorbing, in the long run, 26% of probability mass. Therefore, we observe that convergence largely took place before 2008, whereas the last few years saw more stable patterns or, if any tendency did emerge, it was actually to converge to a state closer to the average.

The values corresponding to this hypothetic future (ergodic) distribution are valid *per se*, but can be complemented by providing information on how fast this state is reached. This information, rarely provided in convergence analysis studies, can be obtained via the transition path analysis or asymptotic half-life of convergence. As indicated by Magrini (1999), this refers to the time it takes to cover half the distance from the ergodic distribution; the results from applying Equation (5) are reported in the three panels (bottom row) of Table 5. A priori, the results might not seem very intuitive, since it takes longer to reach the steady-state during the period leading stronger convergence (2000–2008) than during the second period (2008–2016) of slower convergence. However, it is precisely because the ergodic distribution in Table 5.b is more extreme than in Table 5.c that it actually takes longer to reach it.

**Fig. 2:**
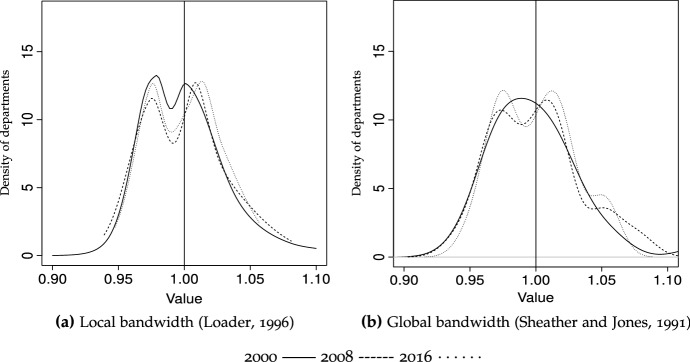
GDP/N (unweighted), densities, 2000 vs. 2008 vs. 2016

**Fig. 3:**
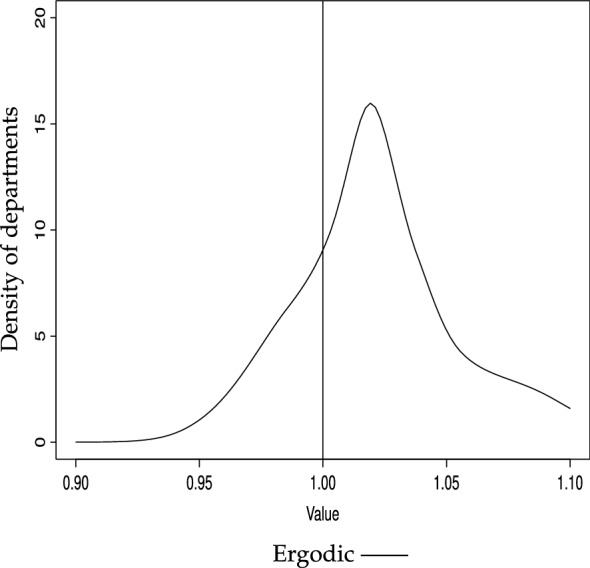
GDP/N (unweighted), ergodic distribution, 2-year transitions Bandwidth: rule of thumb (Silverman 1986)

**Fig. 4:**
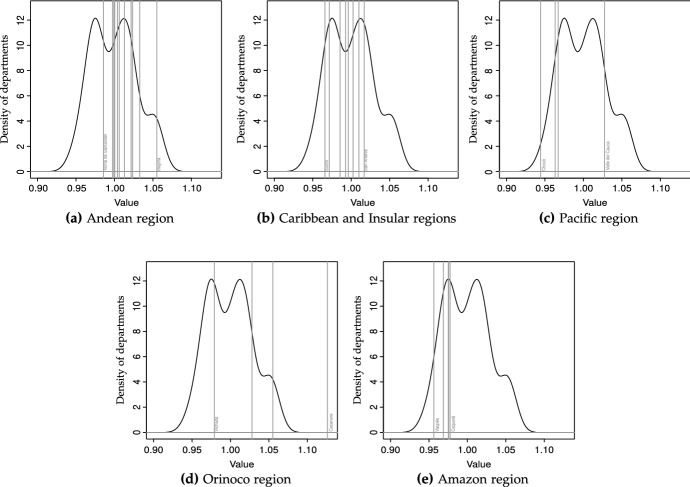
GDP/N (unweighted), densities, 2016 (departments and regions).Notes: The vertical lines in each sub-figure represent the normalized GDP per capita for the departments in each region (with the normalization corresponding to $x_{it}=\text {ln}y_{it}/\text {ln}{\bar{y}}_t$, being $y_{it}$ the per capita GDP of the department). The labels in each sub-figure refer to the poorest and richest departments in each region

The densities estimated nonparametrically corresponding to the initial, final and middle year provide a static view of the distributions whose law of motion is described by the transition matrices. These are depicted in Fig. 2, with solid, dashed and dotted lines corresponding to years 2000, 2008 and 2016. It clearly indicates that the distribution of per capita income was bimodal in 2000, and remained so in 2016, with the probability mass separating further—i.e., the rich become richer. This would confirm that the strong convergence patterns found for pre-2000 years have almost vanished, and that the convergence during our sample period is more strongly related to intra-distribution dynamics (changes in the departments’ relative positions, or churning). Figure 2 actually reports results for different bandwidths, with the one on the left (Fig. 2a) estimated using local smoothing methods, and the one on the right (Fig. 2b) using global (plug-in) selectors. The main differences are attributable to the methods themselves, since local bandwidths take into account how dense is each particular part of the distribution, with the advantage that it can uncover more easily the modes in the vicinity of 1—the national average.^18^ We also depict densities for the different regions for 2016, to get an idea of the multi-modality existing in each region. As shown in Fig. 4, the low number of departments in some regions (particularly the Pacific, Orinoco and Amazon regions) makes it difficult to obtain a clear picture about how many modes could be present. However, Fig. 4 reveals some interesting trends, such as the high contribution of the Amazon region to form the mode corresponding to the poorer departments (see Fig. 4e), the heterogeneity within the Pacific and Orinoco regions, and the contributions of both the Caribbean and Andean regions to create a “middle class”—with the exception of Bogotá (see Fig. 4a).

Will this polarization persist over time? The (discrete) ergodic distributions in Table 5 do not confirm this, since they suggest that probability mass would tend to concentrate in the richer states–regardless of the trends considered (2000–2016, 2000–2008 or 2008–2016). This result is corroborated by the continuous counterpart to the ergodic distributions in Table 5, reported in Fig. 3, which clearly shows that bimodality will vanish, and departments will tend to converge to levels of higher relative per capita income, since the probability mass will become tighter and above unity. However, the upper tail of the distribution will still be fat, indicating that, in the long run, a number of departments will still enjoy per capita income levels well above the average.^19^

We therefore complement the existing literature on regional convergence in Colombia in several ways, by considering a more recent period, and by applying instruments that make the analysis more precise—i.e., the mobility indices, transition path analysis, and the continuous approach to the ergodic distributions. Although some of these instruments had previously been considered, this study is the first to contemplate others in this context, such as the continuous version of the ergodic distributions or the asymptotic half-life of convergence.

As Galvis-Aponte et al. (2017) point out, Birchenall and Murcia (1997) were the first to apply the distribution dynamics model to the Colombian case. These authors considered stochastic kernels as well as some conditioning schemes, but focusing on the 1950–1994 period, for which the patterns are not of convergence. In a later study, Birchenall (2001) also considered the analysis of transition probability matrices and even mobility indices but, unfortunately, the period he analyzed ended in 1995 (so it is difficult to compare), and he concluded that convergence was over in the 1990s. These overall results of lack of convergence hold for studies by other authors such as Ardila Rueda (2004), Branisa and Cardozo (2009a), Gómez (2006), Martínez (2006) and Royuela and García (2015), who have used different tools from the distribution dynamics approach, with the exception of the calculation of ergodic distributions, and rarely mobility indices and half-life of convergence. Some of these studies focus on slightly more recent periods; for instance, the study by Royuela and García (2015) focuses on 1975–2005. However, the overall result for that period persists, i.e., regional per capita income inequalities have persisted until the early 2000s.

The only study focusing on a more recent period, like in our case, is the survey study on regional convergence in Colombia by Galvis-Aponte et al. (2017), which performs some analyses for more recent periods—up to 2016. Although, being a survey study, is not fully comparable to our paper, the conclusions they reach after reviewing the relevant literature suggest that Colombia is not a case of regional convergence (at least in economic terms), and that it is unlikely that lagging regions will close the gap with the richest—at least in the short run. Our results extend and reinforce these findings, since our asymptotic half-life of convergence tells us that, under 2000–2016 trends, although most departments will climb out of the poorest states (1 and 2), it would take 47.874 years to reach halfway to the ergodic distribution (Table 5a). More departments would leave these states under 2000–2008 trends (Table 5b), but it would take them even longer—and it would definitely not be in the short run.

Our results are also compatible (despite focusing on different periods) with some studies such as Bonet and Meisel (2007), who find a process of polarization between Bogotá and the rest of the departments. This polarization is apparent through the unweighted densities estimated non-parametrically (Fig. 2a), which show a bi-modal shape for all periods, with the exception of year 2000 for the global bandwidth (Fig. 2b). The transitions observed in Table 5 are also compatible with this view of polarization, since it is more difficult for the departments in either the poorest or the richest states (states 1 and 5) to abandon them, for all three matrices in Table 5. For the 2000–2016 period, 81% of the poor departments remained in the state of lowest relative-GDP per capita after two years, and that percentage increased to 92% for the richest (state 5). In contrast, for those departments in the second state, the probability of remaining in the same state was 68% (73% and 78% for those in the third and fourth states of relative income per capita).

### Conditioning

#### Weighted analysis

Results for the population weighted analysis are reported in Table 6. As for the rest of the analysis (i.e., mobility indices, transition path analysis and continuous counterparts to the probability matrices), results are presented in the same tables and figures as those corresponding to the unweighted analysis.

Regarding the discrete analysis offered by transition probability matrices in Table 6, results differ remarkably from those obtained for the unweighted analysis. Regardless the period considered (2000–2016, 2000–2008 or 2008–2016), the intra-distribution mobility leads to ergodic distributions with the probability mass overwhelmingly concentrated in the upper states. In several cases, for instance under 2000–2016 trends, the tendency is particularly extreme, with almost 90% of the probability mass concentrated in states 4 and 5. This would suggest that, in the long run, and under current trends, a large share of the population would live in departments with higher GDP per capita. As mentioned above, this does not necessarily mean that within-department inequality declines.

The mobility indices and, in particular, the transition path analysis, which are reported in the bottom rows of Table 6, complement these results—although interpretation is a bit more convoluted. According to the asymptotic half-life of convergence, it would take a much longer period to reach the steady-state when conditioning by population. However, and analogously to what occurred in the unweighted case when comparing the sub-periods, this occurs because the corresponding ergodic distributions are more extreme and, consequently, more difficult to reach.

**Fig. 5:**
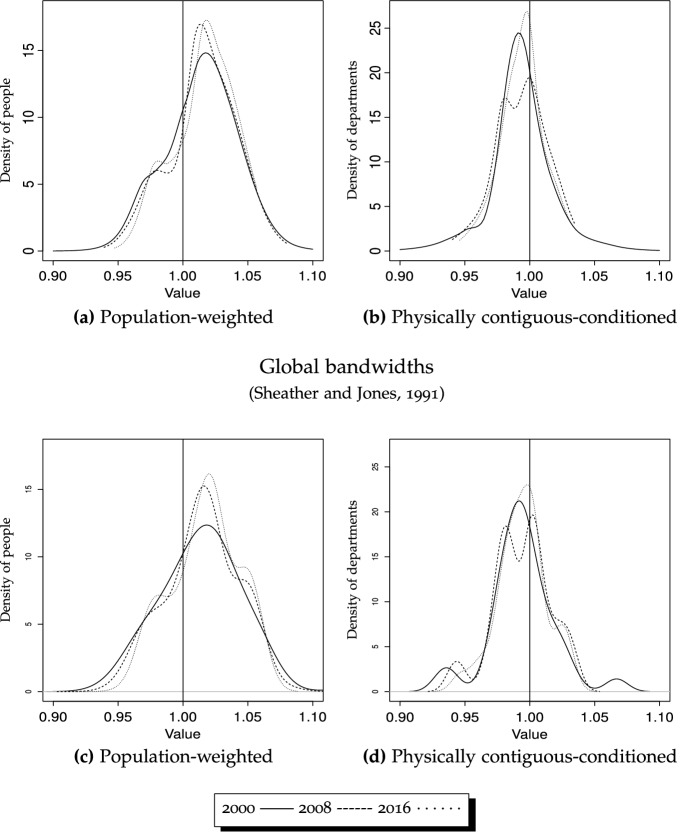
GDP/N (conditioning schemes), densities, 2000 versus 2008 versus 2016

**Fig. 6:**
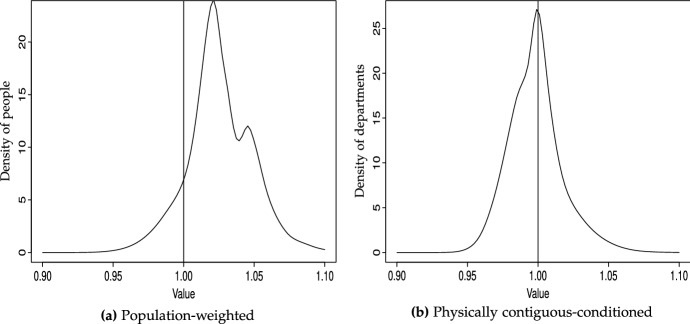
G﻿DP/N (conditioning schemes), ergodic distributions, 2-year transitions. Bandwidth: rule of thumb (Silverman 1986)

The continuous counterparts to the discrete analysis offered by transition probability matrices are reported in Fig. 5a, and in Fig. 6a for ergodic distributions. Results strongly corroborate those tendencies observed when discretizing the normalized per capita income space state as for all years, 2000, 2008 and 2016, bimodality almost disappears. Therefore, comparing years 2000 and 2016 reveals a slight intensification of weighted convergence, although the most prominent feature is the existence of much tighter densities, indicating that when considering population size, discrepancies are much less marked.

**Fig. 7:**
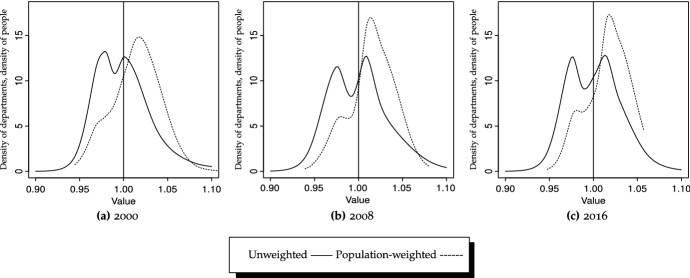
GDP/N, densities, unweighted versus population-weighted Bandwidth: local (Loader 1996)

The importance of weighting is even more obvious when we look at Fig. 7, which provides explicit comparisons between unweighted and weighted distributions for 2000, 2008 and 2016. In all cases the importance of weighting by population is apparent, as densities become much tighter (indicative of more convergence), regardless of the period considered. Finally, as indicated by the ergodic distributions in Fig. 6a, this will ultimately result in strong convergence for people, with probability mass tightly concentrated above unity, although these (weighted) steady-state distributions will become slightly bimodal, with a cluster of people ending up slightly richer than the rest.

#### Conditioning: spatial analysis

The physically contiguous-conditioned (or neighbor-relative) counterparts to the previous analyses—both unweighted and weighted—are reported in Table 7 (transitions, ergodic distributions, mobility indices and transition path analysis), and in Figs. 5b (static densities, local bandwidth), 5d (static densities, global bandwidth), and 6b (ergodic distribution). Analogously to what was found when comparing Table 5 to Table 6, results differ remarkably after conditioning, although several nuances deserve discussion—and are not entirely coincidental as when weighting schemes were introduced. In this case, we observe that intra-distribution mobility differs remarkably for the two sub-periods considered, as it is higher during 2008–2016 (Table 7c)—entries on the main diagonal average to 0.67, compared to 0.76 for 2000–2008 (Table 7b). This finding is corroborated by the mobility indices in Table 7, which also indicate that persistence is lower in the second sub-period ($\mu _1^{2008-2016}=0.680$ and $\mu _1^{2000-2008}=0.634$). These levels of persistence are lower than the state-relative series, which average to 0.81 and 0.77 for the first and second sub-periods, respectively (Table 5).

The implications of disparate mobility levels are not innocuous in terms of long-term distribution, as under 2008–2016 trends probability will be more tightly concentrated above the average, yielding an almost bi-modal ergodic distribution (Table 7c). However, although the results might be partially influenced by the choice of cut-off points,^20^ the overall result is that probability mass tends to concentrate more tightly in states containing values closer to the average—i.e., spatial spillovers exist for Colombian departments.

**Fig. 8:**
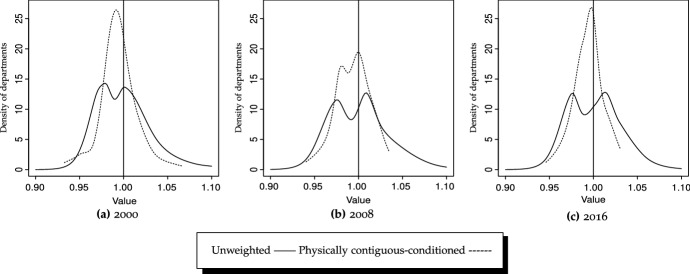
GDP/N, densities, unweighted vs. physically contiguous-conditioned Bandwidth: local (Loader 1996)

Figure 8 reports the physically contiguous counterparts to the unweighted densities (state-relative) in Fig. 2. All three graphics, corresponding to the three periods, show tighter distributions for physically contiguous compared to state-relative per capita GDP series. Therefore, regardless of the choice of cut-off points, each department’s per capita GDP resembles the average of its surrounding departments much more than the average for Colombia. This implies that, for instance, the GDP per capita in Guaviare is much more similar to the average of Meta, Vichada, Guainía, Vaupés and Caquetá than to departments in the Pacific region (Cauca, Chocó, Nariño and Valle del Cauca), thereby corroborating the existence and importance of spatial spillovers. However, the tendency is more marked by the end of the analyzed period, as shown by a much tighter density for 2016 (Fig. 8c). Therefore, the slightly unconditional convergence process is much more accelerated when spatial interactions among neighbors are factored in.

The continuous counterpart to the ergodic distribution in Table 7a is reported in Fig. 6b. It indicates that, under 2000–2016 trends, probability will become tightly concentrated in the vicinity of 1—i.e., departments’ per capita GDP will be very much closer to their neighbors’ average than to the nation’s average.^21^

When will this physically contiguous conditioned ergodic (or stationary) distribution actually be achieved? An approximation is provided by the transition path analysis (asymptotic half-life of convergence) reported in the last rows of Table 7 for the three periods evaluated. The first emerging pattern indicates that, under 2000–2016 trends, the steady state corresponding to neighbor-conditioned relative GDP series would be achieved faster than under either 2000–2008 or 2008–2016 trends. The second pattern shows that the speed is also faster when controlling for geographic spillovers than when these do not enter the analysis—the speed is lower (more years) for the first three rows in the table. There are two explanations for these apparently puzzling results. On the one hand, spatial spillovers had already played a role by the beginning of the period and, therefore, the ergodic distribution is not too far from the initial distribution, at least when compared to the other scenarios. On the other hand, the ergodic distributions corresponding to the physically contiguous case are less extreme and, therefore, can be achieved (hypothetically) earlier.

These results, and especially the trend towards the stratification of provinces in different clubs, are of no minor concern to the authorities, and reveal that there is still some room for policies promoting convergence in per capita GDP among Colombian departments, because the natural tendency towards spatial agglomeration seems to be persistent. Thus, in addition to explicit regional policies and other central government policies to re-balance regional development (central investment projects, endowment of infrastructures, credit policy, etc.), other measures are also needed to balance the tendency towards localization of economic activity induced by market forces. Improvements in the accessibility and the role of market mechanisms in the interior are needed, but increasing the role assigned to official interprovincial migrations is probably necessary too.

**Fig. 9:**
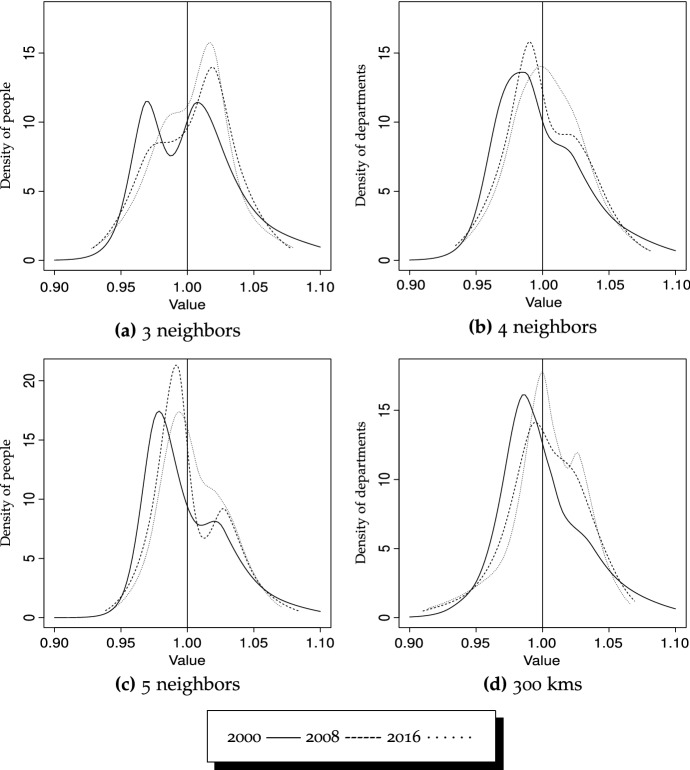
GDP/N, alternative spatially conditioned schemes, densities, 2000 versus 2008 versus 2016

We provide some robustness to this spatial analysis in Fig. 9 of the Appendix. We have computed alternative neighboring criteria (explained in Sect. 3.4) and compare the kernel densities in the initial, intermediate and final year of our sample. It can be observed that in all cases the spatially conditioned distributions are relatively tighter than the original, and distributions are not very different when considering other spatial approaches.^22^ Specifically, the bimodality observed for the unweighted distribution in 2016 (see Fig. 3a), which was virtually smoothed out for the physically contiguous conditioning (see Figs. 5b and d), is also less pronounced for the alternative spatial conditioning schemes (see dotted lines in each panel of Fig. 9).

Again, and similarly to our observations on page 19 regarding the literature on regional convergence in Colombia, comparisons are challenging due to the variety of methods used and periods examined. In this case, the comparative perspective is even more complicated because the previous literature has barely considered in convergence settings how population and space affect the observed tendencies—at least with the proposed methods. Some studies, however, have considered spatial effects—either directly or indirectly. This is the case of Birchenall and Murcia (1997), who propose conditioning stochastic kernels by factoring in the distance to Bogotá; they found that, indeed, physical distance greatly contributed to explain convergence (they also included other variables such as each department’s specialization in mining, and regional exports). These results are compatible with ours, since the spatial conditioning (physical neighbors) yields a much tighter long-run (ergodic) density (Fig. 6b) compared to its unconditioned counterpart (Fig. 3).

Others have also considered, at least indirectly, the relevance of taking into account population and economic mobility across states in Colombia. This is the case of Bonet and Meisel (2007), who find that the persistence of regional inequalities and absence of convergence is partly explained by some policies that have caused increased agglomeration in Bogotá, to the detriment of lagging territories. This strong bimodality effect of Bogotá has also been highlighted by Galvis-Aponte et al. (2017), who find it will take 50 years for most of the Colombia’s 13 largest cities to catch up with Bogotá’s per capita income. These results, as explained in the preceding paragraphs, are also compatible with ours since, as the half-life of convergence in Table 6 indicates, it will take a remarkable amount of time to reach halfway to the ergodic distribution (Table 6a). Therefore, although most of the population will end up living in wealthier territories (regardless of how many people live in them), the existing transitions are not fast enough (the mobility indices are in the vicinity of 0.5–0.6) to accelerate that process.

## Conclusions

The hypothesis of convergence—which (in its simplest form) states that countries’ long-run per capita income levels are independent from initial conditions—has been widely tested over the last thirty years. The issue became particularly important after the emergence of modern growth theory in the mid-1980s, as testing empirically the hypothesis helped to ‘unlock’ the mechanics of economic growth (Johnson and Papageorgiou 2020). This critical role of the convergence hypothesis as a test for either validating or refuting alternative growth theories attracted the interest of many renowned economists (Islam 2003), ultimately leading to a vast increase in the related literature—including several surveys (Durlauf and Quah 1999; Temple 1999; Sala-i-Martin 1996b; De la Fuente 1997; Islam 2003; Johnson and Papageorgiou 2020).

In his informative survey, Islam (2003) attempts to systematize this literature by proposing a classification not only of the different methodologies employed to analyze macroeconomic convergence but also the ways in which it is understood. This is particularly interesting because the first distinction he considers is convergence within an economy vs. convergence across economies, since the latter (regional convergence) has become a large area in itself. As Jerzmanowski (2006) states, “growth experiences differ over time within a country almost as much as they differ among countries”.

In some contexts, these regional disparities have been of particular concern. This is the case of the European Union, for a variety of reasons, including the implementation of cohesion policies, expansion and further integration initiatives, and even the challenge posed by the Brexit, all of which have given rise to a flourishing new body of empirical research. Regional convergence, however, has also been studied in other contexts, including several developing countries. These settings can be even more relevant, as it is now a key global fact that income distribution is more unequal in rapid-growth countries.

In this study we focus on one of these other contexts: Colombia. It has one of the most dynamic and fastest-growing economies in South America, but there is a widespread consensus that it has deficiencies in its distribution of income—including at the regional and departmental levels. Several studies have documented this reality, finding generally either weak or no economic convergence (depending on the period analyzed). The lack of economic convergence in Colombia has become a structural bottleneck, hindering equal opportunities for social and economic development, while simultaneously revealing the poor performance of public policies in providing the right conditions to push regional economies towards a sustainable pattern of economic growth.

We contribute to this literature in several directions. First, our database spans the period 2000 to 2016, enabling us to evaluate the most recently designed and implemented convergence-enhancing public policies. Second, we use the distribution dynamics approach, which has been less frequently used in the case of Colombia, and complement it by also considering mobility indices (Shorrocks 1978), evaluating the asymptotic half-life of convergence (Kremer et al. 2001), and following the ideas suggested by Johnson (2005) in their continuous space-state approach. Third, we adapt the model to control explicitly for the role of demography and geography, introducing a population-weighting scheme, as well as comparing different spatially conditioned GDP series. In this line, as indicated by Partridge and Rickman (2008), although economists tend to argue that the design of policies to alleviate poverty should focus on poor people, therefore supporting worker training and facilitating household mobility, other views highlight the benefits of place-based policies that focus on poor and left-behind places (MacKinnon et al. 2022; Cerqua et al. 2022). Therefore, our variants of the distribution dynamics approach to convergence analysis control explicitly for these theoretical arguments, also highlighted by Royuela and García (2015), revealing the factors that contribute to either accelerate or slow down convergence across Colombian territories.

Results are multiple and can be assessed from several points of view. The unweighted results indicate that, under 2000–2016 trends, convergence will eventually happen, but it will take a very long time, as shown by the high value corresponding to the half-life of convergence. This trend is also revealed by the shape of the densities, bimodal for 2000, 2008 and 2016, a result coincidental with previous literature, despite its analysis of different periods. The ergodic (long-run) distribution is tighter than the static counterparts, and bi-modality will vanish, indicating that convergence will take place, although it is still a long way off. These trends, however, differ when either demographic or geographical conditioning schemes are introduced. For the population-weighted analysis, convergence exists regardless of the sub-period considered. In all cases not only do the ergodic distributions become much tighter, but the bimodality existing in 2000, 2008 and 2016 vanishes almost entirely, implying that more populated departments are improving (and will continue to improve) in terms of income per capita. When taking spatial spillovers into account, (conditional) convergence also accelerates.

Our results are partly in line with previous findings in the literature, since the weak or absent convergence found under the static analysis is corroborated, and our update indicates this pattern still holds. However, the ergodic distributions reveal that, under current trends, most departments will abandon their states of low relative per capita income, catching up with the relatively wealthiest territories. But this will take a long time, as revealed by the asymptotic half-life of convergence. Neither of these instruments have been considered in the previous literature. Shifting the analysis to population-weighted comparisons has obvious implications as the pattern changes completely, indicating that population tends to concentrate in the richest departments and suggesting some possible weaknesses in the cohesion policies. The spatial spillovers, however, were already relevant by the beginning of the analyzed period and their importance will not vanish (Royuela and García 2015). Given its importance, some regions’ wealth might be jeopardized by their geographical proximity to regions in conflict.

In any case, the comparison of our results to previous analyses should be made with caution. The main reason is that our study differs in two key ways: (i) the units of analysis (our study considers the entire population of 33 departments instead of 24 departments prior to the 1991 reform); and (ii) the period (our paper analyzes more recent years than the previous literature, most of which analyzes up to 2005). Moreover, the incorporation of weighting and spatial conditioning in the distribution dynamics approach adds additional sources of discrepancy, which make comparisons with previous studies challenging. In that regard, it should be noted most initiatives for understanding convergence patterns in Colombia covered periods of at least 15 years ago—with the exception of Galvis-Aponte et al. (2017). Therefore, our results provide fresh evidence on the issue and, *a priori*, there is no special reason to expect similarity with previous findings given the novelties in terms of sample and years.

Therefore, although Colombia’s rapid growth has helped to narrow the gap with both Latin American peers and high income countries by accelerating the reduction of poverty rates, several challenges remain, some of which relate to the regional and urban-rural disparities. The poverty rate gaps between the richest and poorest departments have not only persisted but widened, and they are actually higher than in Latin America as a whole and in most of the world. Our analysis has shown that the picture is less dismal when both space and population are factored in. However, as suggested by the transition-path analysis, this will not be a fast process. Therefore, more concerted efforts are needed to alter these dynamics—particularly in terms of higher investment in infrastructures, improving access to public services (and their quality), or commitments to the post-conflict agenda.

## Appendix A: Alternative spatial conditioning schemes

See Fig. 9.

## Footnotes

1 Other contributions also considering social indicators are Branisa and Cardozo (2009b), Aguirre (2005) and Martínez (2006).

2 Our selection is the Gaussian kernel, which is both relatively straightforward to apply and fits most contexts well. Formally, $K(x)=(1/\sqrt{2 \pi }) e ^{-\frac{1}{2} x^2}$. See Li and Racine (2007), among others.

3 Increasing bandwidths for data sparsity can lead to severe bias, essentially because of the kernel being based on a local constant approximation which might suffer from problems in the tails, or trimming of peaks (see Loader 1999).

4 Additional details can be found in Loader (1996, 1999).

5 In their survey, Jones et al. (1996) recommended “solve-the-equation” plug-in bandwidth selectors “as being more reliable in terms of overall performance”.

6 Once each department-year observation has been classified in one of the *K* states, a $5 \times 5$ matrix is built (other popular dimensions are, for instance, $7 \times 7$).

7 This approach can be also understood as a conditional density estimation.

8 See Magrini (1999) and, more generally, Shorrocks (1978).

9 One limitation of this convergence approach should be taken into account, namely, that the data generating process derives from a distribution that is already considering the entire population. However, this approach has been widely used in convergence analyses as a more flexible alternative and has pros and cons with respect to alternative parametric regression-based alternatives. In fact, this limitation is not particularly problematic because the method avoids statistical inference, for which stronger assumptions are needed. Islam (2003) provides a very informative survey on the advantages and disadvantages of each approach to convergence analysis.

10 The department of San Andrés and Providencia is an archipelago and, as such, was excluded from this analysis.

11 It is important to mention that this analysis does not allow us to compute interchangeable effects, which can be better studied in a spatial regression framework. That approach, however, suffers from other limitations. As well as the strong assumptions regarding error behavior in linear regression, the selection of the appropriate spatial model must also be considered. Interpretation of these models is also more complicated.

12 Data on GDP per capita is measured in constant 2010 pesos.

13 We opted to constrain the analysis to the 2000-2016 period for a variety of reasons. Since 2016, uncertainty has increased in the territories affected by the armed conflict despite the signing of the Peace Agreement. The departure of the FARC and the institutional weakness that prevents the state from guaranteeing a secure environment have created the conditions for the emergence of criminal gangs interested in exporting cocaine and illegal mining products. As a result, the territories went from an environment controlled by the FARC to one of criminal gang disputes over economically profitable natural resources, with growing instability, reflected in increased deforestation of the Colombian Amazon, coca crops, and assassinations of union leaders. In addition to this, after the signing of the peace agreements, the Development Plans with a Territorial Approach (PDET) were designed to advance on the agreements of the agrarian reform, and their importance lies in the coordination and governance of resources of the territories to achieve synergies and promote key policies (social property management, infrastructure, health, education, housing, drinking water, basic sanitation, and economic reactivation). The result of the PDET is inconclusive fundamentally because the progress in the fulfillment of the peace process is heterogeneous and fragmented: some regions have progressed more than others, so that this instrument does not always promote convergence toward higher levels of economic and human development in the municipalities most affected by the conflict. If we add to this the difficulties due to the outbreak of COVID-19 in 2020, we can conclude that the trends after 2016 are too unstable from a territorial convergence/divergence perspective and, for the moment, it might be more informative to leave these years out of the study and consider them once some of the uncertainties about the social control of the territory have disappeared. The reports of the Kroc Institute (2021) and PDET (2021) provide detailed information on these lines.

14 The current territorial organization of Colombia was established by the constitutional reform of 1991, which defined 32 departments and the capital city Bogotá, which is a department in itself. Previously, there were only 24 departments.

15 Note that Colombian natural or *geographical* regions do not hold specific powers (they are not administrative units), and in some cases their geographical limits do not coincide with departments’ limits—i.e., some departments are part of different regions. However, given Colombia’s peculiar geography and orography, which both have a critical impact on the development of infrastructures, we consider they help to understand some facts and trends.

16 Similar normalization strategies can be found in, for instance, Sakamoto and Islam (2008). However, in their case, the variable is defined in a slightly different way—as $x_{it}= \text {ln} (y_{it}/{\bar{y}}_t)$. This normalization provides similar information, but we opted to follow our variant in order to avoid negative values, the interpretation of which is slightly less intuitive.

17 This is mainly done in order to minimize the loss of information. It is not common practice, but we consider it makes the same or more sense, particularly due to the advantage of being able to consider relatively short periods—which is the case we are dealing with. In addition, should we be interested in considering not only two-year but also five-year transitions (a popular choice), we would have many more periods available for the analysis, yielding more stable results.

18 We have also tested if the distributions are bi-modal for each of the selected years. For this, we applied the methods based on kernel smoothing proposed by Ameijeiras-Alonso et al. (2021), according to which, considering Sheather and Jones (1991) bandwidths, the number of modes was 2, 3 and 3—for 2000, 2008 and 2016, respectively. These methods, up to now, have not been developed for local bandwidths.

19 Due to the way these continuous ergodic distributions were computed, by calculating $20 \times 20$ transition matrices, and generating uniform distributions for each of these 20 values, it was problematic using either plug-in or local bandwidths, so we finally considered rules of thumb, which are sometimes appropriate alternatives (Silverman 1986).

20 See Temple (1999).

21 The tendency is even sharper for 2008–2016 trends, but it is not reported for reasons of space.

22 We have included only the static scenario in Fig. 9. The replication of all the analyses (transition matrices, ergodic distributions, mobility indices, and half-life of convergence) with the different spatial alternatives can be provided on request.
